# Updated Recommendations from the Advisory Committee on Immunization Practices for Use of the Janssen (Johnson & Johnson) COVID-19 Vaccine After Reports of Thrombosis with Thrombocytopenia Syndrome Among Vaccine Recipients — United States, April 2021

**DOI:** 10.15585/mmwr.mm7017e4

**Published:** 2021-04-30

**Authors:** Jessica R. MacNeil, John R. Su, Karen R. Broder, Alice Y. Guh, Julia W. Gargano, Megan Wallace, Stephen C. Hadler, Heather M. Scobie, Amy E. Blain, Danielle Moulia, Matthew F. Daley, Veronica V. McNally, José R. Romero, H. Keipp Talbot, Grace M. Lee, Beth P. Bell, Sara E. Oliver

**Affiliations:** ^1^CDC COVID-19 Response Team; ^2^Institute for Health Research, Kaiser Permanente Colorado, Denver, Colorado; ^3^Franny Strong Foundation, West Bloomfield, Michigan; ^4^Arkansas Department of Health; ^5^Vanderbilt University School of Medicine, Nashville, Tennessee; ^6^Stanford University School of Medicine, Stanford, California; ^7^University of Washington, Seattle, Washington.

On February 27, 2021, the Food and Drug Administration (FDA) issued an Emergency Use Authorization (EUA) for the Janssen COVID-19 (Ad.26.COV2.S) vaccine (Janssen Biotech, Inc., a Janssen Pharmaceutical company, Johnson & Johnson; New Brunswick, New Jersey), and on February 28, 2021, the Advisory Committee on Immunization Practices (ACIP) issued interim recommendations for its use in persons aged ≥18 years (*1*,*2*). On April 13, 2021, CDC and FDA recommended a pause in the use of the Janssen COVID-19 vaccine after reports of six U.S. cases of cerebral venous sinus thrombosis (CVST) with thrombocytopenia, a rare thromboembolic syndrome, among Janssen COVID-19 vaccine recipients (*3*). Two emergency ACIP meetings were rapidly convened to review reported cases of thrombosis with thrombocytopenia syndrome (TTS) and to consider updated recommendations for use of the Janssen COVID-19 vaccine in the United States. On April 23, 2021, after a discussion of the benefits and risks of resuming vaccination, ACIP reaffirmed its interim recommendation for use of the Janssen COVID-19 vaccine in all persons aged ≥18 years under the FDA’s EUA, which now includes a warning that rare clotting events might occur after vaccination, primarily among women aged 18–49 years. Patient and provider education about the risk for TTS with the Janssen COVID-19 vaccine, especially among women aged <50 years, as well as the availability of alternative COVID-19 vaccines, is required to guide vaccine decision-making and ensure early recognition and clinical management of TTS.

Since June 2020, ACIP has convened 13 public meetings to review data on COVID-19 epidemiology and the potential use of COVID-19 vaccines, including the Janssen COVID-19 vaccine. The COVID-19 Vaccines Work Group, comprising experts in infectious diseases, vaccinology, vaccine safety, public health, and ethics, has held weekly meetings since April 2020 to review COVID-19 surveillance data, evidence for vaccine efficacy and safety, and implementation considerations for COVID-19 vaccines. The work group met three times during the Janssen COVID-19 vaccine pause to review clinical trial and postauthorization safety data for TTS after receipt of this vaccine. The work group also reviewed a risk-benefit assessment of TTS events after receipt of the Janssen COVID-19 vaccine that used an adapted Evidence to Recommendations framework* to guide the assessment. In addition, the COVID-19 Vaccines Safety Technical Work Group, comprising independent vaccine safety expert consultants, held two meetings during the vaccine pause and conducted an independent review of safety data for thromboembolic events that occurred after receipt of the Janssen COVID-19 vaccine. A summary of the data reviewed and work group discussions was presented to ACIP during its emergency meetings on April 14 and April 23, 2021. On April 23, 2021, ACIP voted 10–4 in favor of reaffirming its interim recommendation for use of the Janssen COVID-19 vaccine in all persons aged ≥18 years under the FDA’s EUA. One ACIP member recused herself from voting because of participation in clinical trials or other studies involving companies producing COVID-19 vaccines. After the vote, ACIP members who voted “no” indicated that they would have preferred stronger language regarding the risk for TTS among women aged 18–49 years. All ACIP members agreed that provider and patient education regarding the risk for TTS after vaccination among women aged 18–49 years and awareness of other COVID-19 vaccine options are critical as Janssen COVID-19 vaccination resumes.

TTS is a rare syndrome that involves acute venous or arterial thrombosis and new onset thrombocytopenia in patients with no recent known exposure to heparin.^†^ Although the mechanism that causes TTS is not fully understood, TTS appears to be similar to heparin-induced thrombocytopenia (*4*), a rare reaction to heparin treatment. In the United States, 12 of 15 persons with TTS that occurred after Janssen COVID-19 vaccination had CVST with thrombocytopenia. The clinical presentation of the reported cases among recipients of the Janssen COVID-19 vaccine (which is based on a human adenoviral vector) is similar to that of recently reported cases from Europe after receipt of the AstraZeneca COVID-19 vaccine (which is based on a chimpanzee adenoviral vector), a vaccine that is not authorized for use in the United States (*5*,*6*). All postauthorization U.S. cases occurred among women; one case of CVST with thrombocytopenia occurred in a man, in the 18–49 years age group, during the Janssen Phase III clinical trial. No cases of CVST with thrombocytopenia have been reported after receipt of either of the two mRNA COVID-19 vaccines authorized for use in the United States (CDC, unpublished data, 2021).

As of April 21, 2021, approximately 7.98 million doses of the Janssen COVID-19 vaccine had been administered in the United States. During March 2–April 21, 2021, the Vaccine Adverse Event Reporting System (VAERS) (*7*), the national vaccine safety monitoring system, had received 15 reports of TTS after Janssen COVID-19 vaccination, with clots located in the cerebral venous sinuses and other unusual locations, including in the portal vein and splenic vein, and a combination of venous and arterial thromboses. These 15 reports were confirmed by physician reviewers at CDC and FDA and reviewed with Clinical Immunization Safety Assessment Project investigators,^§^ including hematologists. Thirteen TTS cases occurred among women aged 18−49 years, and two occurred among women aged ≥50 years; no cases postauthorization were reported among men.^¶^ TTS reporting rates to VAERS were 7.0 cases per million Janssen COVID-19 vaccine doses administered to women aged 18−49 years and 0.9 per million to women aged ≥50 years. Among subgroups by age (18–29, 30−39, 40–49, 50–64, and ≥65 years), the reported rate was highest among women aged 30−39 years, with 11.8 TTS cases per 1 million Janssen COVID-19 doses administered. The median age was 37 years (range = 18−59 years), and the median interval from vaccination to symptom onset was 8 days (range = 6−15 days). Certain patients had underlying medical conditions or risk factors for hypercoagulability (e.g., obesity [seven patients], combined oral contraceptive use [two patients], hypothyroidism [two patients], and hypertension [two patients]); no cases occurred among women who were pregnant or had given birth in the previous 12 weeks, and none had a documented history of previous thrombotic events, a known diagnosis of an underlying clotting disorder, or a family or personal history of clotting disorders. None of the patients had any known previous exposure to heparin. All 15 patients were hospitalized, and 12 were admitted to an intensive care unit (ICU). As of the most recent follow-up,** three patients had died, four remained in an ICU, three remained hospitalized (not in an ICU), and five had been discharged home.

While evaluating the evidence to support updated interim recommendations for the use of the Janssen COVID-19 vaccine in the United States, ACIP reviewed a risk-benefit assessment of TTS events after vaccination. This assessment took into account 1) the rate and characteristics of TTS cases; 2) recent COVID-19 epidemiology; 3) modeling and risk-benefit analysis results to quantify COVID-19 hospitalizations, ICU admissions, and deaths prevented with resumption of use of the Janssen COVID-19 vaccine in the United States; and 4) data from jurisdictional COVID-19 vaccination programs regarding whether changes to ACIP recommendations would disproportionately affect certain populations.

The risk-benefit analysis included an assessment of both population- and individual-level risks and benefits. Full details of the analysis methods and results are available (https://www.cdc.gov/vaccines/covid-19/info-by-product/janssen/risk-benefit-analysis.html). The population-level risk-benefit analysis assumed continued use of the mRNA vaccines and estimated the number of COVID-19–related hospitalizations, ICU admissions, and deaths that could be prevented by use of the vaccines; benefits from vaccination were applied to the entire population, both directly to vaccinated persons and indirectly because of herd immunity. The modeling data showed that in 6 months, resuming use of the Janssen COVID-19 vaccine among persons aged ≥18 years at 50% of the prepause administration rate could prevent 3,926−9,395 COVID-19–related hospital admissions, 928−2,236 ICU admissions, and 586−1,435 deaths (depending on assumed future COVID-19 transmission levels), compared with 26 expected cases of TTS (Table 1). Resuming vaccination only among persons aged ≥50 years could prevent 1,361–3,532 COVID-19–related hospitalizations, 295−799 ICU admissions, and 54−257 deaths, compared with two expected TTS cases. The individual-level risk-benefit analysis assessed the risks and benefits of receiving versus not receiving a Janssen COVID-19 vaccine during the 1-month period after the Janssen COVID-19 vaccine pause. For every 1 million doses of the Janssen COVID-19 vaccine administered to women aged 18–49 years, 297 hospitalizations, 56 ICU admissions, and six deaths related to COVID-19 could be prevented, compared with seven expected TTS cases. Among women aged ≥50 years, 2,454 hospitalizations, 661 ICU admissions, and 394 deaths could be prevented, compared with one expected TTS case (Table 2). The benefits (prevention of COVID-19–related hospitalizations and ICU admissions) outweighed the risks (expected TTS cases after vaccination) in all populations. However, the balance of risks and benefits varied by age and sex because cases of TTS were primarily identified among women aged 18–49 years.

**TABLE 1:** Population-level estimated number of and percent decrease in COVID-19–related hospitalizations, intensive care unit admissions, and deaths after resuming use of Janssen COVID-19 vaccine for 6 months* and number of expected cases of thrombosis with thrombocytopenia syndrome, by age group and SARS-CoV-2 transmission level^†^ — United States, 2021

Benefits and harms from resuming vaccination	Resumption strategy				
	Recommended for adults aged ≥18 yrs		Recommended for adults aged ≥50 yrs		
	Low SARS-CoV-2 transmission	Moderate SARS-CoV-2 transmission	Low SARS-CoV-2 transmission		Moderate SARS-CoV-2 transmission
**No. of persons expected to receive the Janssen COVID-19 vaccine**	9.8 million		3.6 million		
**Benefits, no. (% decrease^§^)**					
Hospitalizations prevented	3,926 (1.4)	9,395 (1.6)	1,361 (0.5)		3,532 (0.6)
ICU admissions prevented	928 (1.4)	2,236 (1.5)	295 (0.4)		799 (0.5)
Deaths prevented	586 (1.6)	1,435 (1.8)	54 (0.1)		257 (0.3)
**Harms**					
No. of TTS cases expected	26	26	2	2	

**Abbreviations:** ICU = intensive care unit; TTS = thrombosis with thrombocytopenia syndrome.

* Resumption of vaccination after a 10-day pause that commenced on April 13, 2021. https://emergency.cdc.gov/han/2021/han00442.asp

^†^ This model evaluated the direct and indirect effects of resuming 50% of Janssen COVID-19 administration rates (compared with rate before use was paused) among all adults aged ≥18 years or only among adults ≥50 years compared with not resuming vaccination. The model was also calibrated to both low and moderate COVID-19 transmission levels based on varying assumptions about nonpharmaceutical interventions during the modeled time period.

^§^ Compared with no resumption of Janssen vaccination.

**TABLE 2:** Individual-level estimated number of COVID-19–related hospitalizations, intensive care unit admissions, and deaths prevented after resuming use of Janssen COVID-19 vaccine for 1 month* and number of expected cases of thrombosis with thrombocytopenia syndrome per 1 million vaccine doses, by sex and age group^†^ — United States, 2021

Benefits and harms from resuming vaccination	No. per million vaccine doses administered^§^			
	Females		Males	
	18–49 yrs	≥50 yrs	18–49 yrs^¶^	≥50 yrs
**Benefits**				
Hospitalizations prevented	297	2,454	272	2,821
ICU admissions prevented	56	661	51	760
Deaths prevented	6	394	6	471
**Harms**				
TTS cases expected	7	1	1	0

**Abbreviations:** ICU = intensive care unit; TTS = thrombosis with thrombocytopenia syndrome.

* Resumption of vaccination after a 10-day pause that began on April 13, 2021. https://emergency.cdc.gov/han/2021/han00442.asp

^†^ This analysis evaluated direct benefits and harms, per 1 million Janssen COVID-19 vaccine doses, over 30 days.

^§^ Compared with no resumption of Janssen vaccination.

^¶^ Analyses incorporated one TTS case that occurred in the Phase III trial in a man in the 18–49 years age group.

The summary of evidence showed that the single-dose Janssen COVID-19 vaccine is a highly effective and flexible (e.g., stored at refrigerator temperatures) prevention tool that can be useful in communities with increasing COVID-19 incidence and emerging variants of SARS-CoV-2, the virus that causes COVID-19 (Table 3). Limiting vaccine use to specific populations (i.e., by age or sex) could reduce numbers of TTS cases but could also challenge public health implementation, limit personal choice, and disproportionately affect populations with barriers to vaccine access or who have difficulty returning for a second dose. If the Janssen COVID-19 vaccine were no longer available, excess COVID-19 cases and deaths could occur. Based on this risk-benefit assessment, on April 23, 2021, ACIP reaffirmed its interim recommendation for the use of the Janssen COVID-19 vaccine in all persons aged ≥18 years. This recommendation allows for flexibility, choice, and improved access to authorized vaccine products. ACIP emphasized the importance of providing education for vaccination providers and the public about the risk for TTS and availability of other COVID-19 vaccine options, particularly for women aged 18-49 years.

**TABLE 3:** Summary of risk-benefit assessment for the Janssen COVID-19 vaccine — United States, 2021

Domain	Summary of evidence
Public health problem	COVID-19 cases continue to occur widely throughout the United States; the 7-day average of daily new cases (69,577) is increasing.* During March 1–April 17, 2021, the cumulative COVID-19 incidence among adults was 710.9 cases per 100,000 persons, and the cumulative COVID-19 hospitalization rate for adults was 20.6 per 100,000 persons. Most hospitalizations and deaths continue to occur among adults aged ≥65 years; however, the proportions of hospitalizations and deaths among adults aged ≥65 years are decreasing with increasing vaccination rates in this age group. COVID-19 cases and hospitalizations are increasing in some areas of the country and among younger persons who have not yet been vaccinated. The reasons for these increases might be related to emerging SARS-CoV-2 variants^†^ that are becoming predominant in some communities, as well as reduced use of nonpharmaceutical interventions. Ongoing expansion of COVID-19 vaccination programs is needed to reduce disease incidence among persons who are eligible for vaccination.
Benefits and harms	Benefits and harms were estimated based on CDC models that estimate COVID-19 hospitalizations, ICU admissions, and deaths that could be prevented by continued use of the Janssen COVID-19 vaccine, as well as expected cases of TTS. These models showed that in the upcoming 6 months, resuming use of the Janssen COVID-19 vaccine among adults aged ≥18 years at 50% of prepause administration rates could prevent 928−2,236 ICU admissions and 586−1,435 deaths, compared with an estimated 26 expected cases of TTS. Resuming use of the Janssen COVID-19 vaccine only among adults aged ≥50 years could result in prevention of 295−799 ICU admissions and 54−257 deaths, compared with two expected TTS cases.
Values and acceptability	Values and acceptability were assessed among U.S. adults to understand intent to receive a 1-dose COVID-19 vaccine, change in intent to receive the Janssen COVID-19 vaccine over time, and change in overall vaccine confidence after the Janssen COVID-19 vaccine pause. After the pause was announced, only 37% of respondents considered the Janssen COVID-19 vaccine to be safe, a decrease of 15% compared with the previous 2–3 days.^§^ Willingness to receive the Janssen COVID-19 vaccine decreased from 49% before the pause to 19% as of April 19, 2021, compared with 56%−68% for mRNA vaccines.^¶^ A recent poll suggested that overall intent to be vaccinated has increased, with 40% of respondents reporting they are more likely to receive COVID-19 vaccine now than they were 1 month ago and 36% reporting no change in their intent to be vaccinated.** In contrast, another survey found that since the pause in the Janssen COVID-19 vaccine has occurred, approximately half of respondents who are unvaccinated reported they were less likely to receive a COVID-19 vaccine, regardless of brand (CVS Health, personal communication, April 20, 2021).
Feasibility and equity	Feasibility and equity were assessed based on direct responses to a CDC telephone survey of jurisdictional COVID-19 vaccination programs; the survey included questions on the impact of the pause in Janssen COVID-19 vaccination and implications of possible changes to the Janssen COVID-19 vaccine recommendation. Before the pause, most jurisdictions reported focusing Janssen COVID-19 vaccination efforts on mobile populations or those hard to reach with a second dose, especially persons experiencing homelessness (68%), homebound persons (64%), and those who are involved in the justice system (57%). Jurisdictions reported extensive use of Janssen COVID-19 vaccine in mobile units, hospitals, emergency departments, urgent care settings, and school-based clinics. When asked about populations that would be disproportionately affected by a change in the Janssen COVID-19 recommendation, jurisdictions reported that persons who are experiencing homelessness, homebound persons, those involved in the justice system, and persons working in migrant or seasonal jobs would be the groups most likely to be negatively affected.

**Abbreviations:** ICU = intensive care unit; TTS = thrombosis with thrombocytopenia syndrome.

* CDC. COVID data tracker weekly review. Atlanta, GA: US Department of Health and Human Services, CDC; 2021. Accessed April 22, 2021. https://www.cdc.gov/coronavirus/2019-ncov/covid-data/covidview/index.html

^†^ CDC. SARS-CoV-2 variant classifications and definitions. Atlanta, GA: US Department of Health and Human Services, CDC; 2021. Accessed April 22, 2021. https://www.cdc.gov/coronavirus/2019-ncov/cases-updates/variant-surveillance/variant-info.html

^§^ Frankovic K. Economist/YouGov survey: decision to pause Johnson & Johnson vaccine causes public confidence in vaccine to sink. YouGov. April 15, 2021. https://today.yougov.com/topics/politics/articles-reports/2021/04/15/johnson-johnson-vaccine-confidence

^¶^ SurveyMonkey Research. Poll: demand for J&J vaccine plummets, but vaccine confidence holds mostly steady; data from SurveyMonkey and Outbreaks Near Me. Survey Monkey’s Weekly Research Newsletter. April 20, 2021. https://surveymonkey.substack.com/p/jj-pause

** de Beaumont Foundation. Poll: vaccine confidence grows despite J&J pause. de Beaumont Foundation. https://debeaumont.org/news/2021/poll-vaccine-confidence-grows-despite-jj-pause/

FDA has added a warning to the Janssen COVID-19 vaccine EUA and fact sheets regarding rare clotting events that have been reported among vaccine recipients (*1*). Updated patient education and communication materials reflecting this warning are critical to ensure that women aged <50 years are aware of the increased risk for TTS and that other COVID-19 vaccines are available (i.e., mRNA vaccines) (*8*,*9*). The EUA fact sheet should be provided to all vaccine recipients and their caregivers (as relevant) for careful review before vaccination with any authorized COVID-19 vaccine.

Treatment for TTS that occurs after receipt of the Janssen COVID-19 vaccine is different from the treatment typically administered for blood clots^††^; notably, heparin should not be administered, and consultation with hematology specialists is strongly recommended. A Health Alert Network notification published on April 13, 2021 (*3*) provided additional information and recommendations concerning the identification and treatment of suspected cases of TTS after Janssen COVID-19 vaccination for clinicians, public health officials, and the public. Additional clinical considerations for use of COVID-19 vaccines are available (https://www.cdc.gov/vaccines/covid-19/info-by-product/clinical-considerations.html).

CDC and FDA will continue to closely monitor reports of TTS after receipt of the Janssen COVID-19 vaccine and will bring any additional data needed to guide benefits and risks to ACIP for consideration. The risk-benefit analysis can be updated as needed to reflect changes in the COVID-19 pandemic and additional information on the risk for TTS after COVID-19 vaccination. The ACIP recommendation for use of the Janssen COVID-19 vaccine under an EUA is interim and will be updated as additional information becomes available.

## Reporting of Vaccine Adverse Events

FDA requires that vaccine providers report vaccination administration errors, serious adverse events,^§§^ cases of multisystem inflammatory syndrome, and cases of COVID-19 that result in hospitalization or death after administration of a COVID-19 vaccine under an EUA (*10*). Adverse events that occur after receipt of any COVID-19 vaccine should be reported to VAERS. Information on how to submit a report to VAERS is available at https://vaers.hhs.gov/index.html or 1-800-822-7967. Any person who administers or receives a COVID-19 vaccine is encouraged to report any clinically significant adverse event, whether or not it is clear that a vaccine caused the adverse event. In addition, CDC has developed a new, voluntary smartphone-based online tool (referred to as v-safe) that uses text messaging and online surveys to provide near real-time health check-ins after receipt of a COVID-19 vaccine. In cases of v-safe reports that include possible medically attended significant health events, CDC’s v-safe call center follows up with the vaccine recipient to collect additional information for completion of a VAERS report. Information on v-safe is available at https://www.cdc.gov/vsafe.

**Summary**

**What is already known about this topic?**

On April 13, 2021, CDC and the Food and Drug Administration (FDA) recommended pausing use of the Janssen COVID-19 vaccine after reports of thrombosis with thrombocytopenia syndrome (TTS) among vaccine recipients.

**What is added by this report?**

On April 23, the Advisory Committee on Immunization Practices concluded that the benefits of resuming Janssen COVID-19 vaccination among persons aged ≥18 years outweighed the risks and reaffirmed its interim recommendation under FDA’s Emergency Use Authorization, which includes a new warning for rare clotting events among women aged 18–49 years.

**What are the implications for public health practice?**

Resuming use of the Janssen COVID-19 vaccine will ensure flexibility, choice, and improved access. Education about TTS risk with Janssen COVID-19 vaccine is critical.

## Footnotes

*
https://www.cdc.gov/vaccines/acip/recs/grade/downloads/ACIP-evidence-rec-frame-508.pdf

†
https://brightoncollaboration.us/thrombosis-with-thrombocytopenia-syndrome-interim-case-definition

§
https://www.cdc.gov/vaccinesafety/ensuringsafety/monitoring/cisa/index.html

¶ One case of CVST with thrombocytopenia occurred in a man in the 18–49 years age group during the Janssen Phase III clinical trial.

** As of April 21, 2021.

††
https://www.hematology.org/covid-19/vaccine-induced-immune-thrombotic-thrombocytopenia

§§
https://vaers.hhs.gov/faq.html
